# Early CD4^+^ T cell proliferation and chronic T cell engagement impact myeloma outcomes following T cell engager therapy

**DOI:** 10.1172/JCI192927

**Published:** 2025-07-31

**Authors:** Alyssa M. Duffy, Anshika Goenka, Maryam I. Azeem, Azmain Taz, Sayalee V. Potdar, Sara A. Scott, Ellen Marin, Jonathan L. Kaufman, Craig C. Hofmeister, Nisha S. Joseph, Vikas A. Gupta, Sagar Lonial, Ajay K. Nooka, Madhav V. Dhodapkar, Kavita M. Dhodapkar

**Affiliations:** 1 Department of Hematology/Medical Oncology,; 2 Emory Healthcare, and; 3 Winship Cancer Institute, Emory University, Atlanta, Georgia, USA.; 4 Translational Science and Therapeutics Division, Fred Hutch Cancer Center, Seattle, Washington, USA.

**Keywords:** Immunology, Oncology, Cancer, Immunotherapy, T cells

**To the Editor:** T cell engagers (TCEs) are effective therapies for myeloma but limited by risk of disease relapse and opportunistic infections (1). Optimal TCE dosing schedules have not been defined and the impact of therapy on T cell function in vivo remains unknown. We analyzed biospecimens from myeloma patients receiving teclistamab (2) during step-up dosing (*n* = 28) or ongoing therapy (*n* = 20) (Supplemental Table 1; supplemental material available online with this article; https://doi.org/10.1172/JCI192927DS1), following informed consent approved by the Emory Institutional Review Board (see Supplemental Methods). Step-up dosing led to decline in circulating CD4^+^/CD8^+^ T cells within 24–48 hours in patients with/without major (≥ very good partial response [VGPR] (Supplemental Figure 1, A and B). Step-up dosing also led to a proliferative burst in T cells (Supplemental Figure 1, C and D). CD4^+^ T (but not CD8^+^ T) proliferative burst correlated with tumor response (≥ VGPR,≥ partial response [PR]), event-free survival (EFS), overall survival, and cytokine-release syndrome (CRS) (Figure 1, A–C, and Supplemental Figure 1, E–H). Proliferating CD4^+^ T cells consisted of FOXP3^+^ (T-regulatory-like) and FOXP3^–^ (non-T-regulatory) subsets (Figure 1D). Proliferating non-Treg CD4^+^ T cells had a phenotype of CD28^+^ memory T cells expressing inhibitory checkpoints PD1 and LAG3 (Supplemental Figure 1I). Patients experiencing VGPR or greater had higher relative increase in the non-Treg subset (Figure 1E and Supplemental Figure 1, J and K). FlowSOM analyses of CD4^+^ and CD8^+^ T cells (Supplemental Figure 1, L–O) identified 3 distinct metaclusters (MCs) of proliferating CD4^+^ and CD8^+^ T cells (MC 4,5,6 for CD4^+^ and MC 2,3,4 for CD8^+^). Among CD4^+^ MCs, MC6 matching non-Treg CD4^+^ T cells correlated with response. None of the proliferating CD8^+^ MCs were differentially altered in responders. Nonresponders had higher expression of PD1 in CD4^+^ and CD8^+^ MCs and higher TIGIT and LAG3 in CD4^+^ MCs (Supplemental Figure 2, A–D). None of the baseline T cell MCs correlated with CRS (Supplemental Figure 2, E and F).

A high proportion of circulating T cells remained TCE bound prior to dosing and even after monthly dosing (Figure 1F and Supplemental Figure 3A). Detection of TCE-bound T cells required analysis of freshly isolated T cells and was verified by the detection of TCE isotype (IgG4 for teclistamab) and competition with soluble B cell maturation antigen (Supplemental Figure 3, B–D). Chronic T cell engagement can impact T cell function. Compared to pretreatment samples, T cells from TCE-treated patients exhibited reduced cytokine production following stimulation with anti-CD2/3/28 antibodies or viral peptides and reduced activation/tumor lysis in tumor-T cocultures (Figure 1, G–J, and Supplemental Figure 3, E–H). Posttherapy decline in cytokine production correlated with reduced antitumor function (Supplemental Figure 3I). T cells from posttreatment samples exhibited reduced antitumor effects in vivo following teclistamab injection in humanized mice (Figure 1, K and L, and Supplemental Figure 3, J and K). Both pre- and posttreatment T cells localized to tumor site (Supplemental Figure 3K). However, T cells from posttreatment samples exhibited reduced in situ proliferation (Figure 1L). Reduced T cell function persisted with monthly dosing (Supplemental Figure 3, L and M).

These data demonstrate that outcomes following TCE therapy correlate with in vivo CD4^+^ T cell fitness, evaluable within 48 hours of the first step-up dose, an early timepoint not analyzed in most prior studies (1). Prior studies have emphasized CD8^+^ T cells in TCE outcomes due to their cytolytic capacity (1). Our data suggest that CD4^+^ T cells may also be important as early determinants of TCE efficacy. Early CD4 activation may provide help to other effectors including CD8^+^ T cells. Our data also show that ongoing TCE therapy leads to a decline in function of both CD4^+^/CD8^+^ T cells and loss of TCE antitumor effects in vivo, which may contribute to the observed risk of relapse and infections (3). Inhibitory checkpoints increased in nonresponders support opportunities for future combination therapy. Persistence of T cell dysfunction with current regimens suggest the need to consider earlier and greater dosing frequency deescalation or time-limited dosing to better preserve T cell function in the long term.

## Supplementary Material

Supplemental data

Supporting data values

## Figures and Tables

**Figure 1:**
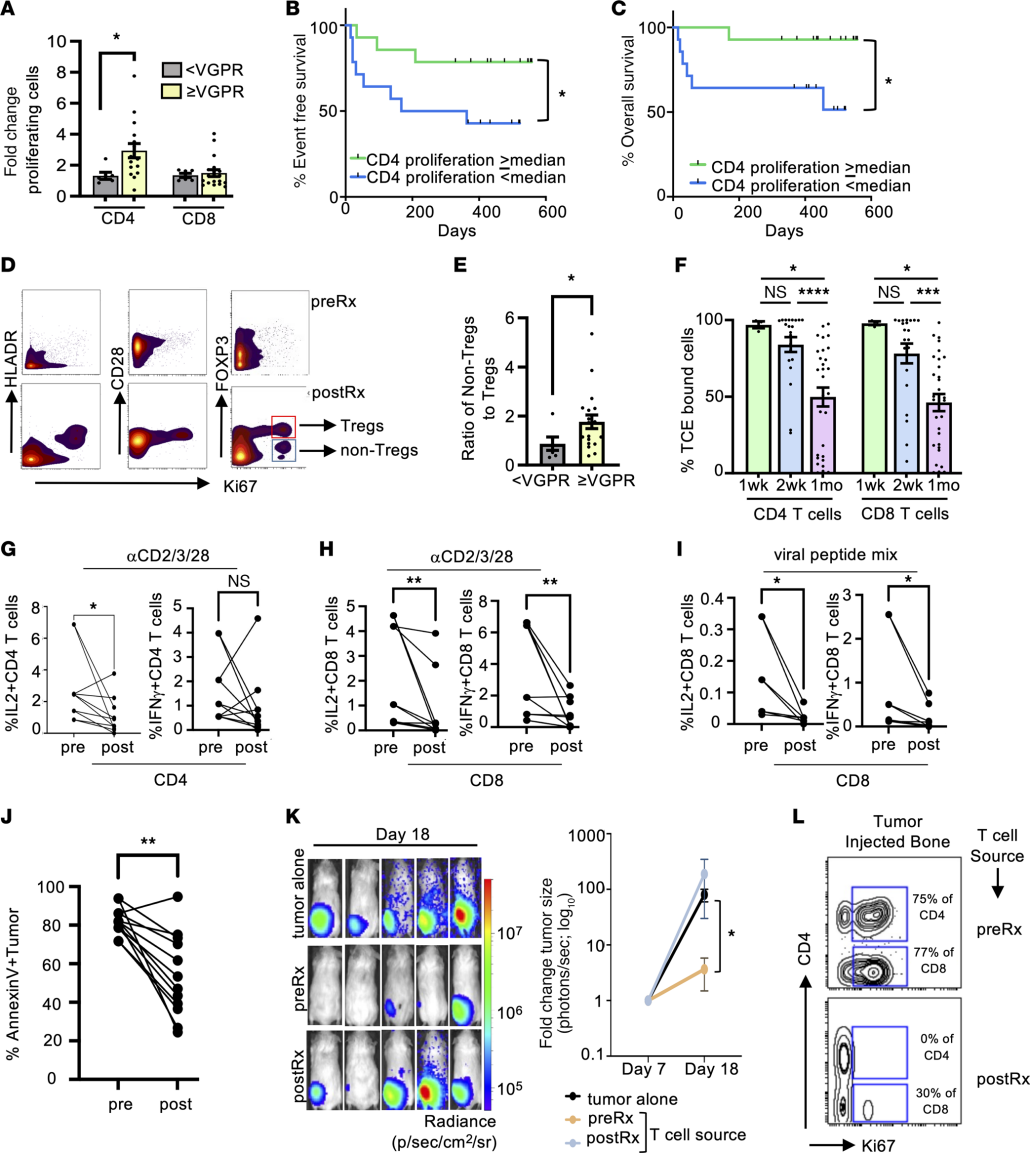
Early CD4^+^ T proliferation and chronic TCE engagement impact outcomes following BCMA-TCE therapy. (**A**) Change in proliferating (Ki67^+^) CD4^+^ and CD8^+^ T cells (fold change postRx versus preRx) in responders (≥ VGPR; *n* = 19) and nonresponders (< VGPR; *n* = 6). (**B** and **C**) Kaplan Meier plots showing event-free survival (**B**) and overall survival (**C**) in cohorts (*n* = 14) based on CD4 proliferation above/below median. (**D**) Expression of HLADR, CD28 and FOXP3 in Ki67^+^ CD4^+^ T cells in a representative patient. (**E**) Ratio of increase in proliferating non-Treg CD4^+^ T cells to Treg CD4^+^ T cells posttherapy in responders (≥ VGPR) and nonresponders (< VGPR). (**F**) % TCE bound circulating CD4^+^ and CD8^+^ T cells in patients (54 samples) receiving weekly (*n* = 3), every 2 weeks (*n* = 21) and monthly dosing (*n* = 30). (**G** and **H**) Proportion of IL2 and IFN-γ secreting CD4 (**G**) and CD8 (**H**) T cells in pre and post therapy (*n* = 10). (**I**) Proportion of IL2 and IFN-γ secreting CD8^+^ T cells pre- and posttherapy specimens following stimulation with viral peptide mix against CMV, EBV, and influenza (CEF) (*n* =7). (**J**) Percent annexin V tumor cells in tumor: T cocultures in pre- and posttherapy samples (*n* = 12). (**K** and **L**) Impact of TCE therapy on in vivo antitumor function. (**K**) IVIS imaging showing tumors at day 18 (d18); Right panel shows fold change in bioluminescence at d18; (**L**) In situ proliferation (Ki-67^+^) of T cells at tumor site (injected bone), as analyzed by mass cytometry. Each dot is an individual sample. Bar graphs show mean ± SEM. *P < 0.05, **P < 0.01, ***P < 0.001, ****P < 0.0001. Mann Whitney was used for statistical analysis in **A** and **E**. (**F**) Kruskall-Wallis with multiple test correction. Wilcoxon matched-pairs signed rank test was used for statistical comparisons in **G**–**J**.

## Version 1 07/31/2025

In-Press Preview

## Version 2 10/01/2025

Electronic publication
