# Supplementary material for: Early CD4+ T cell proliferation and chronic T cell engagement impact myeloma outcomes following T cell engager therapy
Source: J Clin Invest. 2025 Jul 31;135(19):e192927. doi: 10.1172/JCI192927 (PMC12483553; doi:10.1172/JCI192927)
Supplement: Supplemental data [file jci-135-192927-s090.pdf]

Duffy et al.

Supplementary Appendix:

Table of Contents

Supplementary methods

Supplementary text

Supplementary tables

Supplementary figures

Supplementary Methods

Sex as a biological variable
Sex was not considered as a biological variable.

Patients and specimens
Biospecimens were obtained from MM patients receiving teclistamab, a BCMA TCE, both early during step-up dosing (n=28, step-up cohort) or during subsequent therapy (n=20, full dose cohort) following informed consent approved by the Emory Institutional Review Board. For the step-up dosing cohort, patients were hospitalized and biospecimens were collected at baseline prior to dosing and then 24-36h after the stepup dose. For the full-dose cohort, biospecimens were obtained prior to dosing as a part of routine clinical care. Teclistamab dosing was as per package insert and involved 2 step-up doses at least 48h apart followed by first full dose (0.06, 0.3 and 1.5 mg/kg). A risk evaluation and mitigation strategy program was implemented as per FDA guidance. Dosing frequency was reduced to every 2 weeks from cycles 3-6 and further reduced to monthly after 6 cycles in responding patients. Peripheral blood mononuclear cells (PBMCs) were obtained utilizing Ficoll density gradient centrifugation.

Single cell mass cytometry
Freshly isolated or frozen/thawed PBMCs were stained with metal conjugated antibodies following manufacturer's recommendations. Cells were labeled with extracellular antibodies to detect cell surface proteins followed by fixation and permeabilization for intracellular and intranuclear staining using intracellular antibodies. Cisplatin was used to detect cell viability and cells were incubated with intercalator-Ir prior to collection. Data was acquired using the Helios instrument and analyzed with Cytobank analysis software (Beckman Coulter Life Sciences).

Flow cytometry
For cellular phenotyping, cells were washed with PBS and stained for 30 minutes at 4°C using the following markers: CD3 (clone SK7), CD4 (clone RPA-T4), anti-biotin (clone 1D4-C5) (Biolegend), and CD8 (clone SK1) (BD Biosciences). For in vivo studies, mouse cells were gated out using Ter119 (clone TER119) (BD Biosciences), mCD45 (clone 30-F11), and hCD45 (clone 2D1) (Biolegend).

To detect cytokines, cells were first stained with extracellular antibodies for 30 minutes at 4°C using the markers previously described. Cells were washed with PBS and fixed with Cytofix buffer (BD Biosciences) according to manufacturer's recommendations. Following fixation, the following intracellular antibodies were used to detect cytokines: IFNg (clone B27) and IL2 (clone MQ1-17H12f (Biolegend).

Live cells were identified using Live/Dead® Fixable Dead stain from Thermo Fisher Scientific. Samples were washed with cold PBS and acquired on a BDCelesta. Data was analyzed using FlowJo.

Detection of TCE-bound T cells
Freshly isolated PBMCs were incubated with a biotinylated BCMA detection reagent (Miltenyi) for 10 minutes following manufacturer's recommendations. Cells were subsequently stained with an anti-biotin conjugated fluorophore and additional extracellular antibodies for flow cytometry phenotyping as previously described (1).

To alternatively detect bound TCE, cells were stained with anti-human IgG4 (Southern Biotech) based on teclistamab isotype. In order to test competition with soluble BCMA, PBMCs were incubated with 0.001 ug/ml, 0.1 ug/ml, or 1 ug/ml soluble BCMA (R&D Systems) prior to staining for flow cytometry analysis.

Assessment of cytokine production
PBMCs were thawed and rested for 1 hour in cRPMI + 5% pooled human serum prior to stimulation. Cells were stimulated with Immunocult Human CD3/CD28/CD2 T cell activator (StemCell Technologies) or 5 ug/ml per peptide of CEF viral peptide mix (Anaspec) with 1 ug/mL purified anti-CD49d (clone 9F10) and anti-CD28 (clone CD28.2) (Biolegend) in the presence of GolgiStop (BD Biosciences) for 4 hours at 37°C. Detection of cytokines was performed via intracellular flow cytometry, as previously described.

In vitro tumor killing
PBMCs were thawed and cultured with KMS18 cells (gift from Lawrence Boise, Emory) in a 5:1 or 1:1 effector to target ratio. Cells were cocultured in complete RPMI for 24 hours at 37°C in the presence of 10% post treatment patient plasma containing teclistamab. Following incubation, cells were stained with Annexin V (BD Biosciences) according to manufacturer's recommendations and additional extracellular antibodies for analysis via flow cytometry.

In vivo modeling
MISTRG6 mice (1) were intrafemorally injected with $5x10^5$ Oci-My5-Luc MM cells (parental cells gift from Lawrence Boise, Emory). IVIS imaging was performed 7 days post injection to assess tumor growth. Patient PBMCs were thawed, incubated with 1 ug/ml teclistamab for 30 minutes at 37°C, and washed. Following resuspension in sterile PBS, 5 million cells per mouse were retroorbitally injected. After 5 days, mice were dosed with 0.5 mg/kg of teclistamab and re-imaged on day18. Mice were then euthanized and mononuclear cells were harvested from injected/contralateral bone and spleen as described. Cells were then analyzed for human tumor/immune cells using flow or mass cytometry.

Statistical analysis
Cytokine release syndrome was assessed using Lee criteria (1). Myeloma clinical responses were assessed utilizing international myeloma working group criteria (2). Survival was calculated from the time of biospecimen collection, and event-free survival was estimated as time to disease progression or death. Statistical analysis of mass cytometry and flow cytometry was performed using Cytobank and GraphPad Prism. Two-tailed Mann–Whitney was used to compare data between groups.

Study Approval:
Clinical protocol for collection of biospecimens was approved by Institutional Review Board at Emory University. Animal studies were performed in accordance with protocol reviewed by IACUC at Emory University.

Data Availability:
There is no analytic code associated with this manuscript. Supporting data values file included.

Acknowledgments:
This work is the result of NIH funding, in whole or in part, and is subject to the NIH Public Access Policy. Through acceptance of this federal funding, the NIH has been given a right to make the work publicly available in PubMed Central.

MVD is supported in part by funds from NIH, Paula and Rodger Riney Foundation and SCOR award from LLS. KMD is supported in part by funds from NIH CA238471 and AR077926. The authors acknowledge Winship immune monitoring resource supported by P30CA138292.

Author contributions statement:
AMD performed experiments, analyzed data and wrote manuscript.
AG, MIA, TA, SVP performed experiments and analyzed data.
SS, EM extracted clinical data and analyzed data.
NS, CCH, JLK, AN, VG, SN performed clinical research, data analysis and reviewed the manuscript.
KMD and MVD designed and supervised the project, analyzed data and wrote the first draft of manuscript.
All authors reviewed and approved the manuscript.

Competing interest statement:
Authors declare no competing interest relating to this work.

References

1. Dhodapkar KM, Cohen AD, Kaushal A, et al. Changes in Bone Marrow Tumor and Immune Cells Correlate with Durability of Remissions Following BCMA CAR T TRheeferarepnycinesM: yeloma. Blood Cancer Discov. Nov 2 2022;3(6):490-501. doi:10.1158/2643-3230.BCD-22-0018
2. Das R, Strowig T, Verma R, et al. Microenvironment-dependent growth of preneoplastic and malignant plasma cells in humanized mice. Nat Med. Nov 2016;22(11):1351-1357. doi:10.1038/nm.4202
3. Lee DW, Santomasso BD, Locke FL, et al. ASBMT Consensus Grading for Cytokine Release Syndrome and Neurological Toxicity Associated with Immune Effector Cells. Biol Blood Marrow Transplant. Dec 25 2018;doi:10.1016/j.bbmt.2018.12.758
4. Kumar S, Paiva B, Anderson KC, et al. International Myeloma Working Group consensus criteria for response and minimal residual disease assessment in multiple myeloma. *Lancet Oncol*. Aug 2016;17(8):e328-e346. doi:10.1016/S1470-2045(16)30206-6

Supplementary Table1. Patient characteristics

	Step-up cohort (n=28)	Full dose cohort (n=20)
Median Age in yrs (range)	65 (50-86)	66 (48-81)
Sex		
Female	6 (21%)	6 (30%)
Male	22 (79%)	14 (70%)
Race		
White	14 (50%)	6 (30%)
Black	11 (39%)	13 (65%)
Other	3 (11%)	1 (5%)
IgH		
IgA	7 (25%)	4 (20%)
IgG	15 (54%)	10 (50%)
Other	6 (21%)	6 (30%)
IgL		
Kappa	18 (64%)	11 (55%)
Lambda	9 (32%)	9 (45%)
Other	1 (4%)	
Median prior lines of therapy (range)	5 (3-13)	6 (3-11)

Supplementary Fig1

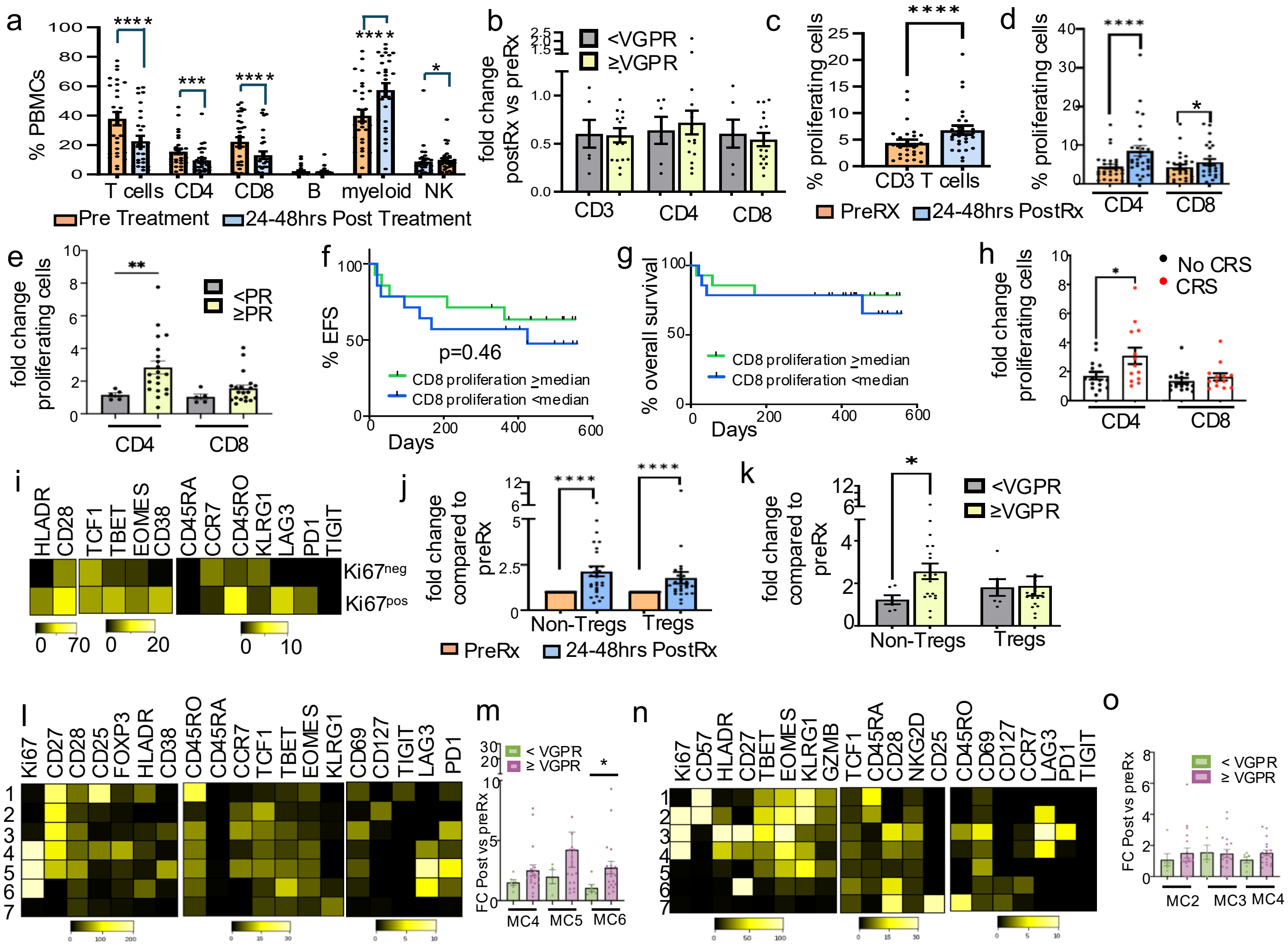

Supplementary Figure1. Immune changes during BCMA-TCE step-up dosing and outcomes

PBMCs collected before and 24-48 hrs following first step up teclistamab dose (n=28) were analyzed using mass cytometry. **a.** Proportions of circulating T, CD4, CD8, B, myeloid and NK cells in paired samples pre/post step-up dose. **b**. Fold change in circulating CD3+, CD4+ and CD8+ T cells in responders (≥VGPR; n=19) and non-responding (<VGPR; n=6) patients. **c-d.** Proportions of proliferating (Ki67+) CD3+T cells (panel c) and CD4/CD8 T cells (panel d) in paired pre and post therapy samples (n=28). **e**. Fold change in proliferating (Ki67+) CD4 and CD8 T cells (fold change postRx compared to preRx) in responders (≥PR; n=20) and non-responders (<PR; n=5) **f-h**. Patients were divided into 2 groups based on fold change postRx in proliferating CD8 T cells either above (n=14) or below (n=14) the median (1.3 fold compared to preRx) for the entire group. Kaplan Meier plots showing event free survival (panel f) and overall survival (panel g). **h**. Bar graph shows change in proliferating CD4 and CD8 cells (fold change postRx compared to preRx) in patients who did (n=13) or did not (n=15) develop cytokine release syndrome (CRS) following teclistamab **i**. Heatmap shows the phenotype of the non-proliferating (Ki67^{neg}) and proliferating (ki67^{pos}) non-regulatory CD4 T cells. **j**. Fold change in proportions of Ki67+ proliferating non-Treg CD4 T cells and Tregs following teclistamab therapy. **k**. Bar graph shows increase in proliferating non-Treg CD4 T cells and Tregs post therapy in responders (≥VGPR; n=19) and non-responders (<VGPR; n=6). **l-o**: FlowSOM (clustering method to build self organizing maps) was used to identify CD4 and CD8 T metaclusters. Ki67 expression was used to identify proliferating cells. **l**. Heatmap shows phenotype of the CD4 metaclusters with 3 distinct proliferating metaclusters (MC4, MC5, MC6). MC4 is CD25 and FOXP3+ consistent with Tregs while MC5 and MC6 are non-Treg proliferating cells. **m**. Fold change in the proliferating CD4MCs 24-48hrs after teclistamab therapy in responding patients (≥VGPR; n=19) and non-responders (<VGPR; n=6). **n**. Heatmap shows phenotype of the CD8 metaclusters with 3 distinct proliferating metaclusters (MC2, MC3, MC4). **o**. Fold change in the proliferating CD8 MCs after teclistamab therapy in responding patients (≥VGPR; n=19) and non-responders (<VGPR; n=6). Each dot is an individual sample. Bar graphs show mean+/- SEM. Wilcoxon matched-pairs signed rank test was used for statistical comparisons in 1a,c,d,e and 1k. Mann-Whitney was used for statistical comparisons in 1b, h, j, k. *p<0.05, ***p<0.001 ****p<0.0001.

Supplementary Fig2

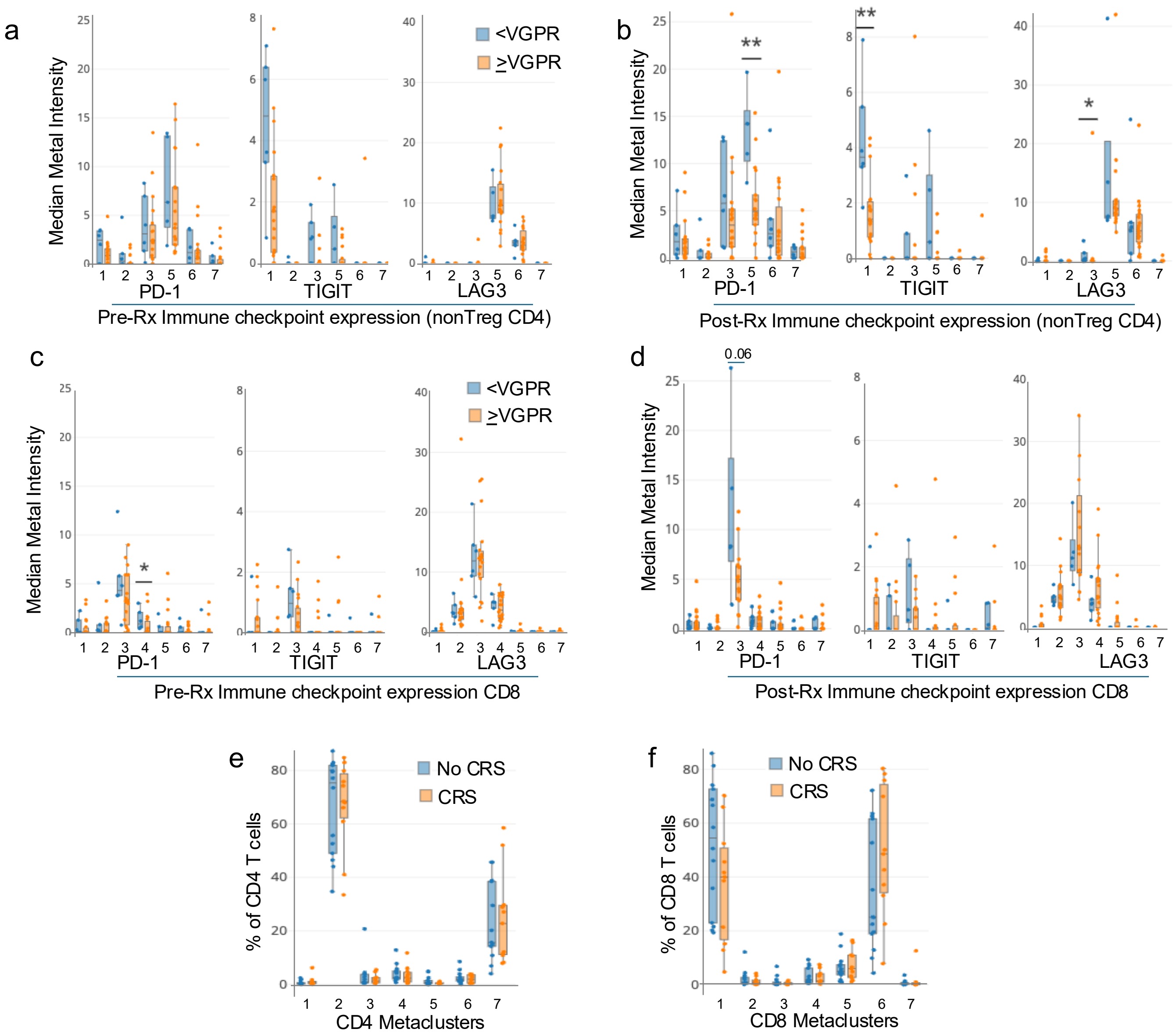

Supplementary Figure2. Correlation between T cell subsets, inhibitory checkpoint expression and development of cytokine release syndrome. FlowSOM was performed on CD4 T and CD8 T cells as shown in supplementary figure 1l-n. **a-b.** Expression of inhibitory immune checkpoints was examined on the CD4 and CD8 metaclusters(MCs)/subsets from responding (≥VGPR; n=16) and non-responding patients (<VGPR, n=6). a-b Expression of PD-1, TIGIT and LAG3 in non-Treg CD4 MCs pre and post teclistamab. **c-d.** Expression of PD-1, TIGIT and LAG3 on CD8 MCs pre and post teclistamab. e-f. Distribution of CD4 and CD8 MCs at baseline pre therapy in patients who did (CRS; n=11) or did not develop (No CRS; n=14) cytokine release syndrome. Each dot is an individual sample. Box plots represent median with Q1-Q3 and error bars as min-max. Mann-Whitney was used for statistical comparisons.*p<0.05, **p<0.01.

Supplementary Fig3

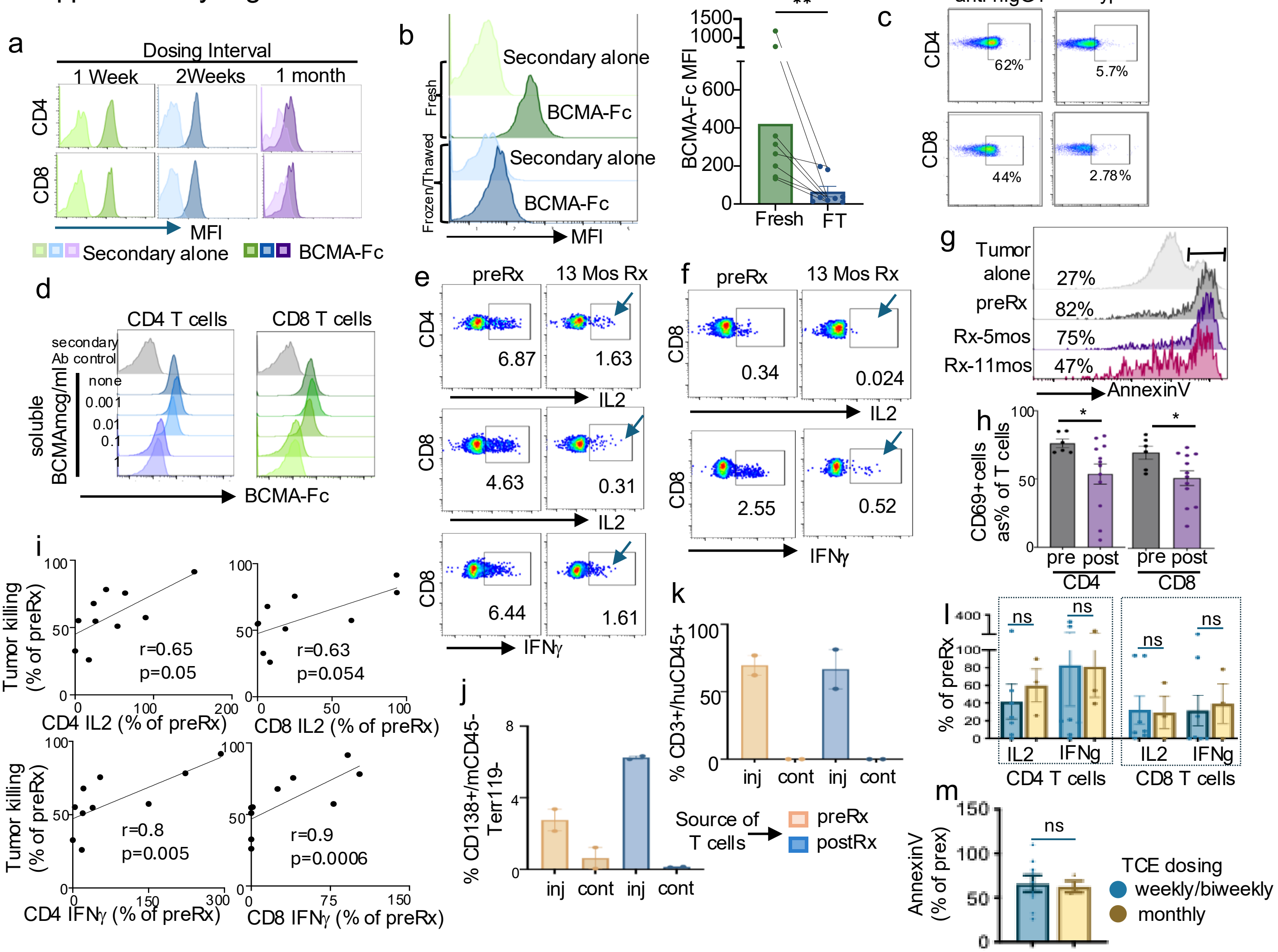

Supplementary Figure 3. Effects of chronic TCE dosing. a-d. Detection of chronically BCMA-TCE bound T cells in pre-dose specimen. **a**. Freshly isolated PBMCs were stained to evaluate TCE binding by flow cytometry. Figure shows mean fluorescence intensity (MFI) of BCMA-Fc compared to MFI for secondary antibody alone as a control in CD4 and CD8 T cells from representative patients for weekly, 2-weekly and monthly dosing intervals. **b**. TCE bound cells were evaluated in paired fresh as well as in frozen thawed samples (n=8). Histogram shows MFI for BCMA-Fc and secondary alone antibody binding in a representative sample when using fresh PBMCs or PBMCSs that were frozen thawed (FT). Dead cells were excluded by using dead cell exclusion dye. Bar graph shows TCE detection in live T cells in paired fresh or frozen thawed PBMC samples demonstrating loss of TCE binding with freeze-thaw. **c**. BCMA-TCE binding to T cells was verified by staining with mouse anti-human IgG4 antibody. Mouse IgG1 isotype was used as control. **d**. BCMA-TCE bound T cells were incubated alone or with increasing doses of soluble BCMA protein for 30 minutes prior to staining with BCMA-Fc. Figure shows dose dependent competition with soluble BCMA. Bar graph in 2b shows mean+/- SEM. Wilcoxon matched-pairs signed rank test was used for statistical comparison,**p<0.01.**e-k. Decline in in-vitro T cell function following chronic TCE therapy.** Paired pre/post-treatment PBMCs were analyzed for secretion of cytokines following TCR stimulation **(e)** and viral peptide mix against CMV, EBV and influenza (CEF) **(f)**. Both plots show representative patient at preRx and 13month on therapy. **g-h**. PBMCs obtained preRx and postRx were examined for their ability to kill BCMA+ MM cells (KMS18) in vitro (effector:target ratio 5:1) in the presence of BCMA-TCE. Increase in CD69 surface expression (**h**) was used as a marker of T cell activation and tumor killing in these cultures was assessed based on AnnexinV+ tumor cells. Histogram shows cell death (AnnexinV+) in tumor cells when cultured alone (tumor alone control), with preRx PBMCs or postRx PBMCs following 5 or 11 months of TCE therapy. **i.** Plots show correlation between tumor killing and the ability of CD4 and CD8 T cells to secrete IL2 and IFNγ (n=10). **j-k. Effects of TCE therapy on in-vivo TCE-mediated redirection**. MISTRG6 mice were injected intrafemorally with MM cells, followed by PBMCs from MM patients before (preRx) or after (postRx) teclistamab therapy. Mice were then injected intravenously with teclistamab to redirect T cells to tumors as described in methods. **j**. detection of hCD138+ tumor cells by flow cytometry in injected (tumor bearing; inj) or contralateral bone (cont); **k**. detection of hCD3+ T cells by flow cytometry in injected/contralateral bone. Figures show data from 2 different mice. Each dot is an individual sample. Wilcoxon matched-pairs signed rank test was used for statistical comparisons *p<0.05. **l-m. Effect of BCMA-TCE dosing interval on T cell function. l.** Decline in IL2 and IFNγ secretion following TCR stimulation in CD4 and CD8 T cells from patients receiving weekly/biweekly BCMA-TCE dose (blue;n=7) and those receiving BCMA-TCE at monthly interval (yellow;n=3). **m**. Decline in ability to kill tumor cells in-vitro by T cells from patients receiving BCMA-TCE at weekly/biweekly (blue;n=9) or monthly (yellow;n=3) dosing frequency. ns=not significant, Mann-Whitney.
